# Combined Micro-PET/Micro-CT Imaging of Lung Tumours in SPC-raf and SPC-myc Transgenic Mice

**DOI:** 10.1371/journal.pone.0044427

**Published:** 2012-09-21

**Authors:** Thomas Rodt, Matthias Luepke, Claudia Boehm, Katja Hueper, Roman Halter, Silke Glage, Ludwig Hoy, Frank Wacker, Juergen Borlak, Christian von Falck

**Affiliations:** 1 Department of Diagnostic and Interventional Radiology, Hannover Medical School, Hannover, Germany; 2 General Radiology and Medical Physics, University of Veterinary Medicine Hannover, Hannover, Germany; 3 Department of Pharmaceutical Research und Medical Biotechnology, Fraunhofer-Institute for Toxicology and Experimental Medicine, Hannover, Germany; 4 Department of Laboratory Animal Science, Hannover Medical School, Hannover, Germany; 5 Institute of Biometry, Hannover Medical School, Hannover, Germany; 6 Institute for Pharmaco- and Toxicogenomics, Hannover Medical School, Hannover, Germany; University of Navarra, Spain

## Abstract

**Introduction:**

SPC-raf and SPC-myc transgenic mice develop disseminated and circumscribed lung adenocarcinoma respectively, allowing for assessment of carcinogenesis and treatment strategies. The purpose of this study was to investigate the technical feasibility, the correlation of initial findings to histology and the administered radiation dose of combined micro-PET/micro-CT in these animal models.

**Material and Methods:**

14 C57BL/6 mice (4 nontransgenic, 4 SPC-raf transgenic, 6 SPC-myc transgenic) were examined using micro-CT and ^18^F-Fluoro-deoxyglucose micro-PET in-vivo. Micro-PET data was corrected for random events and scatter prior to reconstruction with a 3D-FORE/2D-OSEM iterative algorithm. Rigid micro-PET/micro-CT registration was performed. Tumour-to-non-tumour ratios were calculated for different lung regions and focal lesions. Diffuse tumour growth was quantified using a semiautomated micro-CT segmentation routine reported earlier. Regional histologic tumour load was assessed using a 4-point rating scale. Gamma radiation dose was determined using thermoluminescence dosimeters.

**Results:**

Micro-CT allowed visualisation of diffuse and circumscribed tumours in SPC-raf and SPC-myc transgenic animals along with morphology, while micro-PET provided information on metabolism, but lacked morphologic detail. Mean tumour-to-non-tumour ratio was 2.47 for circumscribed lesions. No significant correlation could be shown between histological tumour load and tumour-to-nontumour ratio for diffuse tumours in SPC-raf transgenic animals. Calculation of the expected dose based on gamma dosimetry yielded approximately 140 mGy/micro-PET examination additional to approximately 200 mGy due to micro-CT.

**Conclusions:**

Combined micro-PET/micro-CT imaging allows for in-vivo assessment of lung tumours in SPC-raf and SPC-myc transgenic mice. The technique has potential for the evaluation of carcinogenesis and treatment strategies in circumscribed lung tumours.

## Introduction

A number of genetic disease models of lung cancer have been developed to better understand the molecular causes of this disease. In-vivo imaging in these models could allow a better understanding of the biological processes and allow a time-course assessment of therapeutic approaches [1]–[4]. We report on combined micro-positron emission tomography (PET)/micro-computed tomography (CT) imaging findings in two transgenic disease models of lung cancer. Furthermore, technical considerations and dose issues are addressed.

Both animal models examined here have been reported in the literature [5]–[8].

In the surfactant protein C (SPC)-raf transgenic mouse model overexpression of the the oncogenically activated N-terminal deletion mutant serine-threonine-kinase c-raf-1 BxB to alveolar epithelium is achieved by use of the SPC promoter [5], [6]. Essentially, this targeted overexpression results in adenocarcinomas of the lung, with multifocal adenomatous hyperplasia being defined as the earliest proliferative lesion of dysplastic cells. By the age of 8 month, approximately 60–70% of the lungs have been reported to be tumour, as judged by histopathology [5].

In the SPC-myc transgenic mice the proto-oncogene c-myc is expressed under the control of the SPC promoter [7], [8]. The animals develop multifocal bronchiolo-alveolar hyperplasias, adenomas and carcinomas respectively. At the age of 14.25 months and 9.2 months respectively, 75% of all hemizygous and 80% of all homozygous mice were diagnosed with bonchiolo-alveolar adenocarcinomas [8].

SPC-raf transgenic mice develop multiple small disseminated lung tumours that partially abut another, whereas SPC-myc transgenic mice develop larger circumscribed tumours, i.e. with a well defined interface between aerated lung and tumour.

These two animal models allow probing for mechanisms of carcinogenesis based on genetic cascades that also play a crucial role in the development of adenocarcinoma of the lungs in humans. Furthermore, they offer the unique opportunity to study carcinogenesis in a more realistic setting as compared to models of implanted (xenograft) tumours into immunodeficient mice. In fact, the animals are still immunologically competent, whilst the continuous expression of the transgene secures continuous tumour pressure. Thus, the relevance of overexpressed protooncogenes or disabled tumour suppressor genes can be studied.

Different imaging modalities have been described and their advantages and disadvantages have been discussed for small animal imaging [9]. Micro-CT allows comparatively fast assessment of morphology [3], [10], [11]. Furthermore, metabolic information on the examined tissue can be obtained by the use of other modalities such as micro-PET or optical imaging [9]. The correlation with morphology, e.g. by micro-PET/micro-CT registration, enables exact localization of this metabolic information [12]. More recently, molecular imaging of responsiveness to chemotherapy at the tumour site or imaging of disease candidate genes has become feasible [13], [14].

However, so far only few studies reported on micro-PET of spontaneously developing orthotopic lung tumours as opposed to xenograft models [15]. In this study we report on the feasibility of combined micro-PET and micro-CT imaging in two transgenic disease models of lung cancer and describe the imaging findings. Furthermore, technical considerations and dose measurements are reported and discussed.

## Materials and Methods

### Animals and imaging setup

All animal work followed strictly the Public Health Service Policy on the humane care and use of laboratory animals. Experiments were performed strictly according to a protocol specifically approved by the animal welfare ethics committee of the State of Lower Saxony (No. 33-42502-06/1081, Lower Saxony State Office for Consumer Protection and Food Safety, Germany).

14 C57BL/6 mice (SPC-raf transgenic n = 6, SPC-myc transgenic n = 7 and wildtype n = 1, 10 male, 4 female, see Table 1) were examined with micro-CT and micro-PET. Age at the date of imaging ranged from 261 to 568 days (MV 455, SD 93). Dosage measurements were performed in another 6 mice. Transgenic mice were maintained as hemizygotes in the C57BL/6 mouse strain background. For imaging studies and dose measurements isoflurane inhalation anaesthesia was administered using a nose cone. The animals were placed in prone position on a multimodality bed, allowing changes between imaging modalities without repositioning. A pressure transducer pad was placed under the animal's chest for respiratory monitoring, which was used to control the anaesthesia and for prospective extrinsic respiratory gating.

**Table 1:** Examined animals.

No.	Genetics	Sex	Age (d)	Circumscribed tumour	Tumour ROI_max_/Liver ROI_MV_	Mean histological tumour load (1–4)	Mean Lung ROI_max_/Liver ROI_MV_
1	SPC-raf	M	447			3	1.03
2	SPC-raf	M	447			3.25	2.16
3	SPC-raf	M	261			2	1.32
4	SPC-myc	M	468	Y	5.87	1	0.92
5	SPC-myc	M	468	Y	2.07	1	0.9
					1.19		
6	Wildtype	M	468			1	0.89
7	SPC-myc	F	559			1	1.38
8	SPC-myc	F	559			1	1.73
9	SPC-myc	M	568			1	1.27
10	SPC-raf	M	492			2	1.21
11	SPC-raf	F	492			1	1.63
12	SPC-raf	F	492			1	1.48
13	SPC-myc	M	323	Y	1.82	1	1.2
14	SPC-myc	M	323	Y	1.44	1	1.17

Transgenic status, sex and age at examination are given. In SPC-myc transgenic animals circumscribed tumour was noted, in these cases Tumour ROI_max_/Liver ROI_MV_ is given. For all animals mean histological tumour load and mean Lung ROI_max_/Liver ROI_MV_ are given. Animal 5 had two circumscribed tumours. All SPC-raf transgenic animals have higher Mean Lung ROI_max_/Liver ROI_MV_ values than the wildtype animal.

### Micro-CT and micro-CT quantification

**Figure 1:**
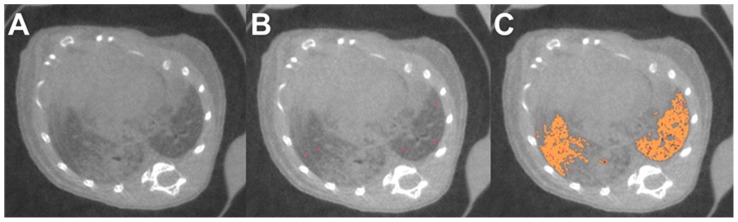
Segmentation of aerated lung volume as a surrogate to assess the multifocal tumor spread in SPC-raf transgenic animals. Micro-CT showing the diffuse bilateral tumour growth (A). Seed points are placed manually in the aerated lung (B). Segmentation of the aerated lung is performed applying a region growing algorithm (C).

Respiratory gated micro-CT was performed (GE Explore Locus, 80 kV, 450 µA, General Electric Healthcare, Chalfont St. Giles, UK) with an effective isotropic voxel size of 0.094 mm. The scan field of view (FOV) was 32.8 mm. For respiratory gating the respiratory signal from the pressure transducer pad was used to generate the image acquisition time points using the software Biovet (m2m Imaging Corp., Cleveland OH, USA). Images of the chest and adjacent structures were reconstructed and calibrated to the Hounsfield scale. For quantification of the multifocal tumours in SPC-raf transgenic animals a region growing segmentation of the aerated parts of the lungs using 20–40 seed points with a threshold tolerance of 2% was used as a surrogate, as direct measurement was not feasible (Figure 1). The segmentation routine and evaluation of the method has already been reported in detail [10].

### Micro-PET, coregistration and micro-PET measurements

Micro-PET (GE Explore Vista, General Electric Healthcare, Chalfont St. Giles, UK) was performed using ^18^F-Fluorodeoxyglucose (^18^F-FDG), (Department of Nuclear Medicine, Hannover Medical School, Hannover, Germany). Prior to the examination the animals fasted for 8 hours. 10 MBq of ^18^F-FDG were injected into the tail vein and data acquisition was started 45 minutes after the administration. A static acquisition was performed for 30 minutes. Images were corrected for random events and scatter prior to reconstruction with a 3D-Fourier rebinning/2D-ordered-subsets expectation-maximization (3D-FORE/2D-OSEM) iterative algorithm. Reconstructed voxel size was 0.39×0.39×0.76 mm.

Rigid registration of the micro-CT data and the micro-PET data was performed using anatomical landmarks with the software MicroView 2.3 (General Electric Healthcare, Chalfont St. Giles, UK). Anatomical landmarks were the tip of the left cardiac ventricle, the lateral border of the right ventricle, anterior and posterior pole of the spleen and the dorsal maximum of the spinal convexity.

For measurements of tracer uptake regions of interest (ROIs) were placed in four different lung regions (upper and lower quadrant on the right and the left side), the left ventricle, the liver and circumscribed tumours. ROI volume in mm^2^, average count rate and standard deviation were acquired as well as minimum (ROI_min_) and maximum (ROI_max_) count-rates. Tumour-to-non-tumour ratios were calculated for the four lung quadrants as the maximum count-rate ratio of the lung region and the mean count rate of the liver (Lung ROI_max_/Liver ROI_MV_). Furthermore, the tumour-to-non-tumour ratio for circumscribed lesions in SPC-myc transgenic mice was calculated as the maximum count-rate ratio of the lung tumour and the mean count rate of the liver (Tumour ROI_max_/Liver ROI_MV_). The mean ROI size was 3.5 mm^3^ for the lung quadrants, 11.9 mm^3^ for the liver and 11.6 mm^3^ for lung lesions.

### Histology

Micro-CT and micro-PET images were correlated to necropsy and histology in all cases. Two days after micro-CT and micro-PET examination animals were sacrificed. Lungs were excised, filled with Tissue-Tek O.C.T.® (Sakura, Finetek Europe, NL) by intratracheal injection and subsequently fixed in 4% buffered formalin (pH 7.2). After dehydration (Shandon Hypercenter, XP) lungs were embedded in paraffin. Sections (2 µm thick) were deparaffinized with xylene and hematoxylin and eosin (H&E) stained. Histological scoring (1–4: 1 = no tumour; 4 = complete obstruction of the area by tumour) was performed for the four lung regions to assess the regional tumour load.

### Statistical Analysis

An independent samples t-test was performed to investigate the significance of group differences between Tumour ROI_max_/Liver ROI_MV_ and Lung ROI_max_/Liver ROI_MV_ for SPC-myc transgenic animals. Furthermore, a Welch-test was performed due to the different variances. Correlation of Tumour ROI_max_/Liver ROI_MV_ and histology was tested using a Spearman correlation in SPC-raf transgenic animals. P<0.05 was considered as statistical significant.

### Dose measurements

In this study dose measurements of the micro-PET component were performed in 6 separate mice using thermoluminescence dosimeters (TLD). Extensive micro-CT dosage measurements for various protocols were performed in earlier studies using the same experimental setup and have been published [16]. LiF: Mg,Cu,P TLD rods of 6 mm length and 1 mm diameter (TLD-100H, Thermo, Waltham, MA, USA) were used for the measurements, which were calibrated using an irradiation reference dose of 540 µGy. The reference dose referred to the radiation quality of ^137^Cs (662 keV). At 511 keV the response of the TLD is just 3% lower. Therefore, no correction was made with respect to the radiation energy. As the TLD were very sensitive to contact, handling was performed using a vacuum forceps. A special container was used for transportation. In order to protect the TLD during irradiation, they were packed into polyethylene tubes (length: 15 mm, diameter: 1.1 mm), which were sealed at the end. The irradiated TLD were analyzed with an automatic TLD reader 5500 (Thermo).

**Figure 2:**
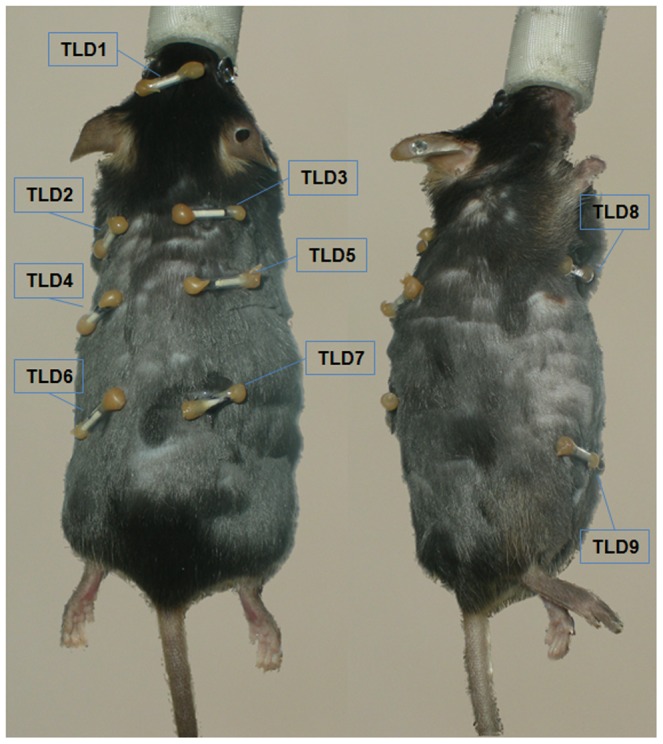
Placement of TLD on anaesthetized mouse. A: Cranial view showing TLD positions 1–7 (see table 2). B: Lateral view showing TLD positions 8 and 9 (see Table 2).

The TLD were placed on the anesthetized animals in 9 positions (see Figure 2, table 2). Following intraperitoneal administration of 10 MBq ^18^F-FDG the animals were kept anesthetized for 45 minutes. Afterwards, static micro-PET measurement was performed for 30 minutes. After the imaging procedure, the TLD were removed from the animals and analyzed.

**Table 2:** TLD positions 1–9 and corresponding dose mean values and standard deviation 75 minutes after intraperitoneal administration of 10 MBq ^18^F-FDG.

Position	Description	Mean dose	SD
		[mGy]	[mGy]
1	Head (dorsal)	2.1	0.3
2	Left hemithorax (dorsal)	2.9	0.5
3	Right hemithorax (dorsal)	2.7	0.6
4	Left thoracoabdominal junction (dorsal)	3.5	0.7
5	Right thoracoabdominal junction (dorsal)	3.5	0.8
6	Left abdomen (dorsal)	4.3	1.6
7	Right abdomen (dorsal)	4.3	0.7
8	Thorax (ventral)	3.9	0.7
9	Abdomen/bladder region (ventral)	12.4	8.2

### Dose estimation

The dose received by a mouse during a micro-PET scan cannot be measured directly. The positrons, resulting from the ^18^F-decay, have such a low range that they hardly leave the body of the mouse. The dose can therefore only be estimated or calculated with Monte-Carlo methods. To validate the Monte Carlo data in this study, the proportion of the dose caused by gamma radiation was determined.

### Dose caused by positrons

The average positron energy of ^18^F is 0.2428 MeV and the branching ratio for positron emission is 96.73% [17]. The average range of the positrons is about 1.5 mm, so that almost all the kinetic energy of the positrons is absorbed in the body of the mouse. The complete absorbed energy in the mouse results from the average energy per positron and the number of positrons emitted in the body of the mouse.

The number of emitted positrons results from the number of decays, which depends on the biological and physical half-life. For all further assessments and comparisons, it is assumed that the excretion is negligible, so that the radionuclide decays completely in the mouse (i.e., infinitely long exposure time):
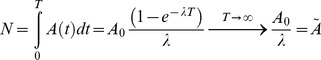
(1)

N: number of decays
A(t): activity
A_0_: administered activity
Ã: cumulated activity
T: exposure time
λ: decay constant of ^18^F

The total absorbed energy divided by the mass of the mouse results in the energy dose:
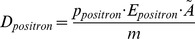
(2)

D_positron_: dose caused by positron radiation
p: branching ratio for positron decay (p = 0.9673)
E_positron_: average energy of positrons
m: mass of the mouse

### Dose caused by gamma radiation

In calculating the dose caused by gamma radiation, it must be considered that in a mouse only about 5% of the radiation is absorbed [18].
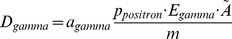
(3)

D_gamma_: dose caused by gamma radiation
a_gamma_: absorbed fraction of gamma energy
E_gamma_: energy of gamma photons (2 · 511 keV)

The sum of the two doses D_positron_ and D_gamma_ results in the total dose of the mouse.

### Measured dose

The dose in a mouse caused by gamma radiation can only be estimated, as doses measured by the TLD are doses outside the mouse. The doses of the TLD and the mice are dependent on the dose rate and the exposure time. The dose is as follows:
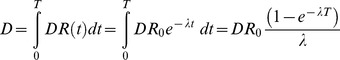
(4)

D: Dose
DR(t): time-dependent dose rate
DR_0_: dose rate at the time of intraperitoneal injection
T: exposure time
λ: effective decay constant of ^18^FDG

As the exposure time for the TLD is shorter than for the mice, the dose of the TLD has to be corrected. For the correction we assume the following: 1. The dose in the mouse is determined only by physical decay. There is no elimination. 2. The spatial distribution of the dose rate within the animal should remain approximately constant during exposure time, i.e. the enrichment of ^18^F-FDG in the bladder is not considered. The dose in the mouse can be derived from equation 4. The slight attenuation of radiation through the mouse tissue is corrected by the factor a_gamma_.
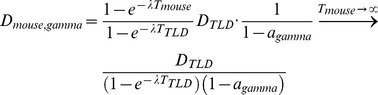
(5)

T_TLD_: exposure time of TLD (75 min)
T_mouse_: exposure time of mouse

To compare the TLD values with the theoretical values the exposure time of the mouse is set to infinity, i.e. it is assumed that the ^18^F completely decays in the body.

## Results

### Micro-CT

No adverse reactions due to anaesthesia occurred. Image acquisition took approximately 20 minutes for respiratory gated micro-CT. Micro-CT allowed visual assessment of high-contrast anatomical structures to high detail, but had limitations in soft tissue differentiation. Respiratory gating visually resulted in good image quality especially near the diaphragm where severe respiratory motion artefacts would have occurred. However, the imaging time increased compared to 14 minutes for an ungated exam, depending on the respiratory rate.

### Micro-CT/micro-PET Coregistration

The quality of rigid coregistration of micro-PET and micro-CT was visually assessed. Registration of landmarks with the highest fiducial registration error was repeated when the alignment of borders was considered insufficient. Coregistred data allowed for the placement of the ROIs for micro-PET measurements based on both micro-PET and micro-CT morphological information.

### Micro-PET Images

**Figure 3:**
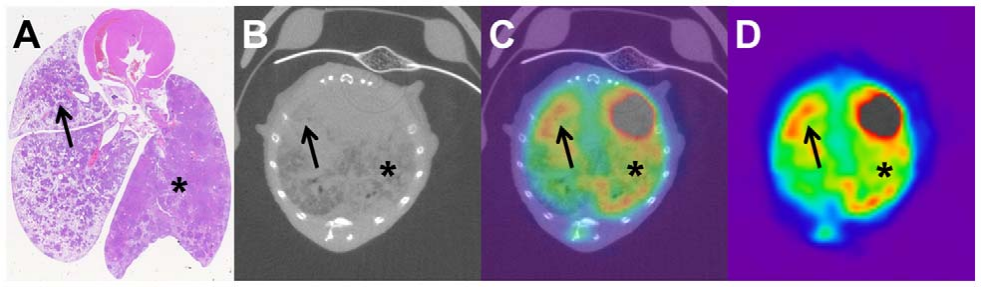
Correlation of histology to micro-CT and micro-PET. Advanced tumor stage in SPC-raf transgenic animal (A–D). Histology (A) and micro-CT (B) show diffuse tumor growth. Micro-CT/micro-PET registration (C) and micro-PET show slight diffuse increased glucose metabolism (D). Asterisk and arrow indicate corresponding lung areas in histology, micro-CT and micro-PET.

**Figure 4:**
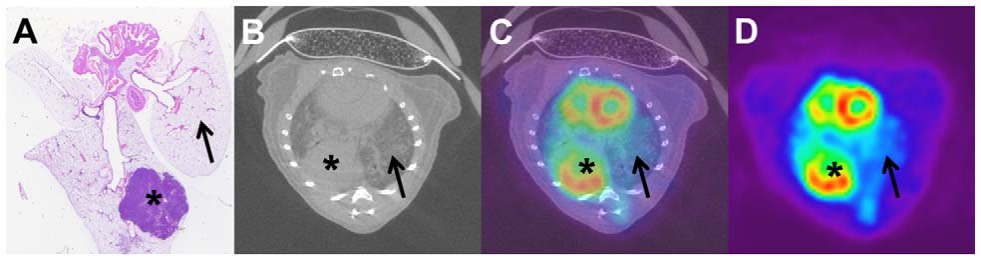
Correlation of histology to micro-CT and micro-PET. Advanced tumor in SPC-myc transgenic animal (A–D). Histology (A) and micro-CT (B) show focal tumor growth. Micro-CT/micro-PET registration (C) and micro-PET show the focal increase of glucose metabolism (D). Asterisk and arrow indicate corresponding lung areas in histology, micro-CT and micro-PET.

10 MBq of ^18^F-FDG were injected into the tail vein without technical problems in all animals of this study. Micro-PET allowed for the visualization of metabolic activity (Figure 3 and 4). Circumscribed tumour regions in SPC-myc transgenic animals showed an increased glucose metabolism. Tumour ROI_max_/Liver ROI_MV_ was 2.47 for circumscribed lesions. No significant difference was detected between Tumour ROI_max_/Liver ROI_MV_ and Lung ROI_max_/Liver ROI_MV_ for SPC-myc transgenic animals (p = 0.003 t-test, p = 0.220 Welch-test).

In the normal animal histological assessment scores were 1 for all 4 lung regions. Lung ROI_max_/Liver ROI_MV_ ranged from 0.70 to 1.08 (MV 0.89, SD 0.16). The Lung ROI_max_/Liver ROI_MV_ of all 4 regions ranged from 0.90 to 2.44 (MV 1.47, SD 0.40) in SPC-raf transgenic animals. Histologic assessment scores ranged from 1 to 4 for all 4 lung regions (MV 2.09, SD 0.90). No significant correlation of Tumour ROI_max_/Liver ROI_MV_ and histology was found using a Spearman correlation in SPC-raf transgenic animals.

### Dose estimation

The dose caused by positrons is given by equation 2. With an administered activity of 10 MBq and an average mass of 30 g for a mouse a dose of 119 mGy results. To calculate the dose caused by the gamma radiation according to equation 3, the absorbed fraction of gamma radiation in the body of the mouse must be determined. Monte Carlo calculations yield a value of 4.4% for mice of 20 g and a value of 5.5% for mice of 40 g. A value of 4.9% for mice with a mass of 30 g was computed by linear interpolation. Therefore, the dose caused by gamma radiation amounts to 24.5 mGy and the total calculated dose to 143 mGy.

### Dose measurements

The TLD were removed from the mice 75 minutes after intraperitoneal injection. However, the ^18^F-FDG was at that point only partially decayed (radioactivity elimination half-life of 110 minutes, renal elimination of up to 20% of ^18^F-FDG in humans was neglected in this study).

**Figure 5:**
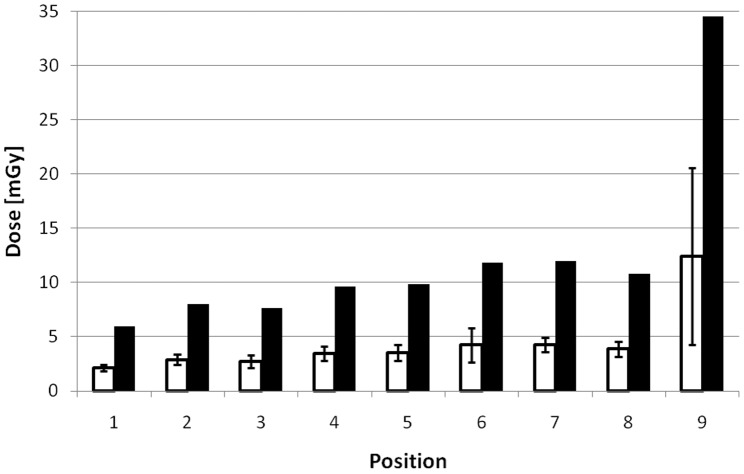
Results of TLD-measurements at different positions (mean doses with standard deviation). Designations of positions according to table 2. White columns: measured dose of the TLD, black columns: calculated dose for the mice according to equation 5.

The results of the TLD measurements and the calculated doses according to equation 5 are shown in Figure 5. The doses in positions 2 to 8 (trunk) are very similar. The lowest dose is measured in position 1 (head). The highest dose is found in position 9 (bladder). This value also has the highest standard deviation. The dose values given do not differentiate between tumour areas and healthy organs.

## Discussion

So far only few studies reported on micro-PET of spontaneously developing orthotopic lung tumours in murine animal models. In this study we showed the feasibility of a combined micro-PET/micro-CT approach for the analysis of tumour metabolism in SPC-raf and SPC-myc transgenic mouse models and report initial imaging findings and dose estimations.

We examined only one wild-type animal in this study, therefore this feasibility study is limited by the missing control group. However, the wild type animal could exemplify the results expected in a negative control, showing no consolidation in micro-CT and no increased glucose utilization in micro-PET. In future studies the Mean Lung ROI_max_/Liver ROI_MV_ values in transgenic animals as compared to wild-type animals could be examined.

There were no adverse side effects noted. Although the technical demands are still challenging we were able to show that the method can be performed as a routine diagnostic procedure and therefore could be used for follow-up studies. Critical steps of the procedure were especially the administration of the radioactive tracer by injection into the tail vein. This procedure proved to be challenging in previous experiments, as part of the tracer may be injected beside the vein. Alternatively, intraperitoneal injection or injection via an intravenous catheter can be performed [19], [20].

Registration of micro-CT data to micro-PET data is another crucial part of the procedure that can prove difficult even though the position of the animal is kept unchanged between the different modalities due to a multimodality bed that can be used for imaging and transfer of the animal from one modality to the other. The reported anatomical landmarks were helpful to achieve good registration results although different techniques should be further investigated in the future, as precision of registration is crucial due to the minuteness of the anatomical structures. Precision of registration further influences the achievable precision of ROI placement for PET measurements. However, as we measured Tumour ROI_max_/Liver ROI_MV_ in this study we believe the registration effect on the measurements should be rather small. Alternative registration techniques could include extrinsic methods such as artificial external landmarks or intrinsic methods such as mutual information techniques [21], [22]. Alternatively, registration issues could be overcome by the use of dedicated small animal PET-CT-scanner where both modalities are incorporated in the same machine [23].

As the accuracy of the registration of micro-PET to micro-CT further depends on the accuracy of the morphological background information in micro-PET and micro-CT, prospective respiratory gating was applied in this study. This technique has been reported to improve image quality especially in the lung areas near the diaphragm [24]. The beneficial effects of more sophisticated gating techniques such as e.g. intrinsic gating have been reported [25]–[28]. Another technique to increase morphological background information could be the administration of contrast agents, which was not performed in this study.

It has been demonstrated that ^18^F-FDG-uptake is closely linked to the pathologic grading [12]. The examined bronchiolo-alveolar adenocarcinoma of the lung show a comparatively good histological differentiation. This may explain the moderate increase in Tumour ROI_max_/Liver ROI_MV_ for circumscribed lesions in the SPC-myc transgenic animals. In the SPC-raf transgenic animals the small size and the development of adenocarcinoma via adenomatous hyperplasia are likely reasons why this study did not show significant correlation of Lung ROI_max_/Liver ROI_MV_ and histological tumour burden. A link between lesion size and degree of differentiation (and subsequent ^18^F-FDG-uptake) has been demonstrated in previous small animal imaging studies [12].

Correction for partial volume effects or calculation of standardized uptake values were not performed in this study, as it was primarily designed to assess the technical feasibility and potential radiation exposure when performed as part of a routine follow-up exam. Furthermore, the estimation of the standardized uptake value is known to be quite sensitive to errors in the measurement of the bodyweight and the injected dose.

Dose calculations based on the Monte Carlo method have been performed for the administration of ^18^FDG in mice [17], [18]. A dose of 140 mGy was calculated for a 30 g mouse with an administered activity of 10 MBq assuming that no elimination takes place. This result shows a good accordance to the result (143 mGy) of our estimation. However, we underline that our calculations are an estimate as some assumptions as stated above cause imprecision.

Radioactivity accumulation, as seen in the images and measured using TLDs, was seen mostly in the heart, kidneys and bladder, as found in Monte Carlo simulations (see table 2). The bladder is exposed to most of the activity. The results of our gamma radiation measurements can be grouped for different body regions. The chest received a dose of 8.8 mGy, the abdomen 12 mGy and the pelvic region 35 mGy. The calculated value of 24.5 mGy is within this dose range of 9 to 35 mGy, confirming the calculated value by the measurements.

All these values demonstrate that micro-PET significantly contributes to the total radiation dose an animal is exposed to in follow-up studies including micro-CT (usually about 150–250 mGy/micro-CT scan, depending on the protocol and gating technique used) and micro-PET. Measurements using the micro-CT protocol applied in this study yielded an average dose of 202 mGy [16], 143 mGy for micro-PET thus accounted for approximately 41% of the total dose/combined micro-PET and micro-CT examination in this study.

In previous studies we already discussed that significant adverse effects on follow-up studies due to these radiation doses are unlikely under the assumption of reasonable scan intervals and follow-up periods [16]. Stochastic effects are not likely to affect study results in most cases due to the time needed to reach an effect. Elevated gene expression has been reported after radiation doses of 20–500 mGy and might influence molecular genetic analysis [29]. However, deterministic effects such as structural changes to the lungs, changes of lung function or cardiac function or unintended therapeutic radiation effects are unlikely given the reported LD_50/30_ of 7.52 Gy and a reported threshold dose of 11 Gy for significant elevation in lung density [30], [31].

To maintain a comparable signal-to-noise ratio to imaging studies in humans a comparatively high dose is necessary in small animals due to the minuteness of voxels. Alternative techniques that can be performed without radiation exposure include optical imaging techniques that have been reported to allow imaging of metabolism and molecular mechanisms applying a variety of different tracers. However, to obtain the morphological background information micro-CT might still have to be performed. Furthermore the PET technique has some advantages compared to optical imaging including the very high sensitivity and the better potential with regard to translational research issues. Magnetic resonance imaging can provide morphological and functional information, however limitations due to susceptibility artefacts have to be considered for the assessment of the lungs.

### Conclusions

In conclusion, we showed that combined micro-PET/micro-CT imaging allows in-vivo assessment of lung tumour metabolism and morphology in SPC-raf and SPC-myc transgenic mice. The technique has potential for the evaluation of carcinogenesis and treatment strategies in circumscribed lung tumours, although technical limitations currently exist and larger numbers of animals have to be examined to further establish and evaluate the use of quantitative approaches in these animal models. The administered radiation dose of the micro-PET component added to the micro-CT component – although high compared to human imaging – should allow the use of the technique for longitudinal studies without direct adverse effects caused by the radiation.
